# Comparison of three generic quality-of-life metrics in peripheral arterial disease patients undergoing conservative and invasive treatments

**DOI:** 10.1007/s11136-019-02166-0

**Published:** 2019-03-30

**Authors:** Svenja Petersohn, Bram L. T. Ramaekers, Renske H. Olie, Arina J. ten Cate-Hoek, Jan-Willem H. C. Daemen, Hugo ten Cate, Manuela A. Joore

**Affiliations:** 1 Department of Clinical Epidemiology and Medical Technology Assessment (KEMTA), Maastricht University Medical Centre +, Maastricht, The Netherlands; 2 School for Public Health and Primary Care (CAPHRI), Maastricht University, Maastricht, The Netherlands; 3 Department of Biochemistry, Cardiovascular Research Institute Maastricht (CARIM), Maastricht University, Maastricht, The Netherlands; 4 Department of Internal Medicine, Maastricht University Medical Centre +, Maastricht, The Netherlands; 5 Department of Vascular surgery, Maastricht University Medical Centre +, Maastricht, The Netherlands

**Keywords:** Peripheral arterial disease, Peripheral revascularisation, Utility, EuroQol EQ-5D (EQ-5D), SF-6D

## Abstract

**Purpose:**

To determine the effect of revascularisation for peripheral arterial disease (PAD) on QoL in the first and second year following diagnosis, to compare the effect depicted by Short Form Six Dimensions (SF-6D) and EuroQoL five Dimensions (EQ-5D) utilities, and Visual Analogue Scale (VAS) scores and to analyse heterogeneity in treatment response.

**Methods:**

Longitudinal data from 229 PAD patients were obtained in an observational study in southern Netherlands. Utility scores were calculated with the international (SF-6D) and Dutch (EQ-5D) tariffs. We analysed treatment effect at years 1 and 2 through propensity score-matched ANCOVAs. Thereby, we estimated the marginal means (EMMs) of revascularisation and conservative treatment, and identified covariates of revascularisation effect.

**Results:**

A year after diagnosis, 70 patients had been revascularised; the EMMs of revascularisation were 0.038, 0.077 and 0.019 for SF-6D, EQ-5D and VAS, respectively (always in this order). For conservative treatment these were − 0.017, 0.038 and 0.021. At 2-year follow-up, the EMMs of revascularisation were 0.015, 0.077 and 0.027, for conservative treatment these were − 0.020, 0.013 and − 0.004. Baseline QoL (and rest pain in year 2) were covariates of treatment effect.

**Conclusions:**

We measured positive effects of revascularisation and conservative treatment on QoL a year after diagnosis, the effect of revascularisation was sustained over 2 years. The magnitude of effect varied between the metrics and was largest for the EQ-5D, which may be most suitable for QoL measurement in PAD patients. Baseline QoL influenced revascularisation effect, in clinical practice this may inform expected QoL gain in individual patients.

## Introduction

Peripheral arterial disease (PAD) is a chronic disease, characterised by the atherosclerotic narrowing of the lower extremity arteries [1]. PAD prevalence is estimated to be 3–10% overall, and 15–20% in the population older than 70 [2]; these numbers seem to be increasing [3]. The disease spectrum ranges from asymptomatic PAD to limb and life-threatening acute leg ischemia [4]. Symptomatic PAD, characterised by exercise-induced occurrence of ischemic muscle pain, causes loss in quality of life (QoL) through reduced physical well-being, mobility, independence and capacity to handle everyday life [5]. Peripheral revascularisation, the open- or endovascular restoring of blood flow in the legs, (f.i. angioplasty, bypass surgery), is typically applied for acute limb ischemia or disease progression despite conservative treatment [2] to restore peripheral reperfusion and reduce the symptom burden.

Previous studies have shown a positive effect on QoL a year after revascularisation [6]; the long-term effects of revascularisation are less verified as progression of atherosclerosis can cause restenosis [7]. Studies showed that 6 months after revascularisation, mean EuroQoL five Dimensions (EQ-5D) utilities increased, then stagnated during the following year [8]; 4 years after revascularisation, pain was the only Nottingham health profile domain significantly improved [9]. This calls into question the sustainability of the effect of revascularisation on QoL.

Guidelines recommend revascularisation only in selected patients with mild to moderate disease [7]. This indicates that disease severity might be a covariate of revascularisation effect on QoL, and some patients might achieve more desirable results than others. This hypothesis is supported by studies showing that 1 year after revascularisation, a proportion of patients did not achieve the desired results: 24.4%, 30.8% and 21.0% of patients did not have improved SF-36 domain scores for physical function, pain or a relevant EQ-5D utility improvement, respectively [10, 11].

In the above-mentioned studies, different methods were used to generate preferences for QoL. The Short Form 36 Health Survey (SF-36) and EQ-5D are based on the valuation of hypothetical health states by members of the general public, i.e. general public preference, in contrast the Nottingham health profile uses the patient’s self-perceived health state preference, i.e. patient preference. It is acknowledged that different methods to generate QoL estimates will measure different aspects of QoL and thus will result in similar but not identical estimates. Research on instruments using general public vs. patient preferences has shown that results can differ, with the general public valuing health worse than patients do [12]. These findings have been confirmed in the valuation of cardiovascular events [13]. All mentioned instruments are generic, i.e. not designed specifically for PAD patients but can be used in any patient population. Differences can also arise between two generic, general public-based instruments [14], and arguments for and against several generic instruments in PAD patients have been presented [15–18]. The review of Poku et al. [18] concludes that the evidence on the psychometric properties of QoL instruments in PAD patients was limited and did not allow for the detection of superiority of one instrument. The evidence focussed on construct validity and responsiveness and reported favourable results for both SF-6D and EQ-5D. The review of Dyer et al. [19] positively commented on the convergent validity and responsiveness of the EQ-5D in PAD patients but did not assess the SF-6D.

PAD treatment is not curative but targeted at relieving PAD symptoms. Consequently, sustainability of QoL gains after revascularisation and variability in the magnitude of gains by patient characteristics are relevant factors in clinical decision making. Beyond that, however, estimations of treatment effect on QoL directly affect the number of quality-adjusted life years attributable to that intervention, and thus play a key role in the evaluation of cost-effectiveness of PAD treatment. Differences between QoL instruments can influence cost-effectiveness estimates, which can misinform policy decision and eventually can lead to the suboptimal use of healthcare. To address those issues, we (1) evaluated, 1 year after PAD diagnosis, the effect of revascularisation on QoL in terms of magnitude and influence of covariates, and compared these results between three QoL metrics, (2) evaluated, 2 years after PAD diagnosis, the sustainability of the effect of revascularisation in year one on QoL, in terms of magnitude and influence of covariates and compared these results between three QoL metrics. This paper presents estimates of treatment effect and offers recommendations for the choice of QoL metric.

## Methods

### Study design

This observational study was conducted between January 2009 and November 2013 in three Dutch hospitals. Approval was obtained at the Medical Ethical Committee (CMO) of the MUMC+. Medical history and QoL was documented in consecutive newly diagnosed PAD patients, who were followed up over 2 years with repeated QoL measurements and documentation of peripheral revascularisation interventions.

### Study population

Patients referred to the vascular department for newly diagnosed PAD were eligible for participation. Inclusion criterion was an ankle brachial index (ABI; the ratio between systolic blood pressure in ankle and arm, measured at rest [20]) of < 0.9 in any leg, measured in the hospital. Patients were included after signing informed consent. Exclusion criteria are listed in Appendix 2. Furthermore, patients were excluded from the analysis when none of the baseline and follow-up QoL instruments had been returned. To ensure homogeneity of time since revascularisation, patients were excluded when revascularisation took place less than 90 days before year 1 follow-up, this was based on medical expert opinion.

### Data collection

For each patient, a case report form was created in an online database, containing patient characteristics, QoL and treatment. Patient characteristics were self-reported in an interview with a research nurse or study physician. At baseline, 1 and 2 years after study inclusion, patients filled in the SF-36 and the EQ-5D measurement instruments. By questionnaire, patients reported treatments received and cardiovascular events experienced during the previous year, 1 and 2 years after baseline (see Appendix 2 for a definition of cardiovascular events); these data were cross-checked with patient medical files for completeness. A research nurse telephoned the patient upon missing data or ambiguous answers.

#### Patient characteristics and treatment

A summary of patient characteristics tested as covariates of treatment effect, their definitions and specifications used in the analyses is given in Table 1. Patients received conservative treatment according to PAD guidelines [7]. This included lifestyle advice regarding smoking cessation and physical exercise, and pharmacotherapy focussed on controlling blood pressure and cholesterol levels. Patients were advised to do unsupervised exercise or received exercise therapy supervised by a physiotherapist. Invasive treatment was defined as peripheral revascularisation which entailed endovascular interventions (e.g. angioplasty with and without stent placement) and open surgery (e.g. atherectomy and endarterectomy, and bypass surgery). Revascularisations were considered relevant for this study when performed within 1 year of PAD diagnosis.

**Table 1:** Names and definitions of patient characteristics

Characteristic	Definition
Disease severity	
Fontaine stage	PAD severity grading system [21] Mild = (I) asymptomatic, (IIa) claudication at > 200 m walking distance Severe = (IIb) claudication at < 200 m walking distance, (III) rest pain and (IV) necrosis or gangrene
ABI	Lower ABI (left or right ankle blood pressure/higher brachial blood pressure) [22]
Claudication distance	Distance walked in m to provoke claudication symptoms
Rest pain	Patient-reported pain at rest
Complaints in daily life	Patient-reported complaints during activities of daily life
Progressive symptoms	Patient reported, within the past 6 months
Demographics	
Age	In years
Gender	Male or female
BMI	Body mass in kg divided by the square of the body height in m [23]
Currently smoking	Patient-reported smoking status
Comorbidities	
Stroke	Diagnosis of stroke or transient ischemic attack > 6 months ago
Myocardial infarction	Diagnosis of acute myocardial infarction > 6 months ago
DM I	Diagnosis of insulin-dependent diabetes
DM II	Diagnosis of non-insulin-dependent diabetes
Hypertension	BP of > 140/90 mmHg and treated with antihypertensive medication
Hypercholesterolemia	Treatment with cholesterol-lowering drugs
Elevated D-Dimer	In patients ≤ 50 years old: D-Dimer > 500 µg/L In patients > 50 years: D-Dimer in µg/L > patient’s age * 10
Impaired kidney function	Indicated by Modification of Diet in Renal Disease estimated glomerular filtration rate (MDRD). Estimated from serum creatinine, age and gender Cut-off: < 60 ml/min/1.73 m^2^ [24]
Malignancies	Previous or current malignancies

#### Short Form 36 Health Survey based SF-6D

The SF-36 is a well-known generic health-related quality-of-life (HR-QoL) metric that has been extensively tested in Dutch populations [25]. The SF-6D has been developed to estimate HR-QoL using ten of the thirty-six items of the SF-36 [26]. Four to six ordinal answers are offered per item, each answer matched with a preference weight to value the desirability of the answer. In the absence of a Dutch tariff, the UK tariff of the SF-6D was used. Combining the valued item responses, domain scores and an overall utility are calculated, each of them between 0.29 and 1.00 to indicate maximum disability to perfect health [25].

#### EuroQoL five dimensions

The EQ-5D is a generic QoL instrument. Since 2008, the 3-level version of the EQ-5D used in this study is the preferred QoL measure in economic evaluations conducted for NICE in the United Kingdom [27]. In the Netherlands, this recommendation has been superseded in favour of the newer 5-level version of the EQ-5D in 2016 [28].The instrument consists of two metrics, the first being a self-classification of health in five domains: mobility, self-care, usual activities, pain/discomfort and anxiety/depression. The respondent indicates if ‘no problems’, ‘some problems’ or ‘severe problems’ occur in each domain; the Dutch tariff of Lamers et al. [29] is used to value the response with a preference weight. All domains combined, a utility is created; the maximum utility of one indicates perfect health, a utility of zero indicates death and the minimum utility of -0.33 indicates conditions worse than death [30].

The second metric, the Visual Analogue Scale (VAS) is a psychometric response scale, recording the respondent’s valuation of their overall health on a scale from 100 to 0, representing best imaginable to poorest imaginable health [30]. The VAS represents a patient’s preference for her own health state. For comparability purposes, VAS scores were divided by 100 to create a score between 1 and 0.

### Missing data

To prevent a loss of precision and the introduction of bias through the exclusion of patients with missing data, missing items of the quality-of-life instruments and baseline patient characteristics were replaced using multiple imputation [31]. Categorical items of the QoL instruments were imputed using dummy coding [32]. We set the number of imputations to 10 and performed sensitivity analysis comparing outcomes of the pooled imputed datasets to a complete case analysis (see Appendixes 1 and 4). Patients who died received a score of 0 in all following QoL measurements.

### Propensity score matching

For each of the 10 imputed datasets, a propensity score (PS) was estimated using logistic regression of baseline patient data [33]. The propensity score was created by testing all baseline patient characteristic parameters for their ability to predict treatment assignment, selecting those parameters with the highest C-statistics and adding parameters that remained unbalanced until the propensity score resulted in adequate covariate balance of baseline characteristics. On this score, each revascularised patient was matched (with replacement) with one conservatively treated patent using the nearest neighbour technique and a calliper of 0.2 [34]. Covariate balance after matching was assessed by comparison of patient characteristics in the treatment groups and by means of visual inspection of QQ plots and PS distributions in the original and matched groups [34]. PS-matched datasets are adjusted against confounding by indication of treatment, allowing outcomes of treatment groups to be compared. PS matching was performed in R version 3.3.3.

### Statistical analysis

Characteristics of patients with complete and incomplete QoL measurements were compared using Bonferroni corrected *t* tests and Chi-square tests [35]. Paired-samples *t* test were used to compare baseline QoL scores of the three instruments. Scatterplots and Pearson correlations were used to explore the effect of time since revascularisation on QoL change at year 1 follow-up.

To explore covariates of treatment effect and compare QoL response in revascularised and conservatively treated patients, analysis of covariance (ANCOVA) was used in the matched cohort producing estimated marginal means (EMMs) of revascularisation and conservative treatment in a post hoc analysis. Patient characteristics described in Table 1 and their interaction terms with revascularisation were included into the models. A backwards deletion approach with the *P* value set to 0.05 was used; all variables were tested for multi-collinearity, variables were excluded if variance inflation factor (VIF) > 1/(1–model *R*^2^) [36]. Variables found significant in one of the three QoL metric’s models were entered into the models of all metrics. The analysis was conducted on baseline to year 1 change and baseline to year 2 change, and the latter analysis excluded patients with revascularisations in the second year. Analysis results that could not be pooled across multiple imputation datasets were presented as ranges. Sensitivity analyses were performed by comparing EMMs to crude scores and by applying the ANCOVA models in:

1. the unmatched sample;
2. the unmatched sample, exclusively using patients without cardiovascular events during follow-up;
3. the unmatched sample, exclusively using complete cases;
4. a sample excluding patients revascularised in the second half of the first follow-up year.

All statistical analyses were conducted on SF-6D, EQ-5D and VAS for comparison, using IBM SPSS Statistics version 23.

## Results

**Fig. 1:**
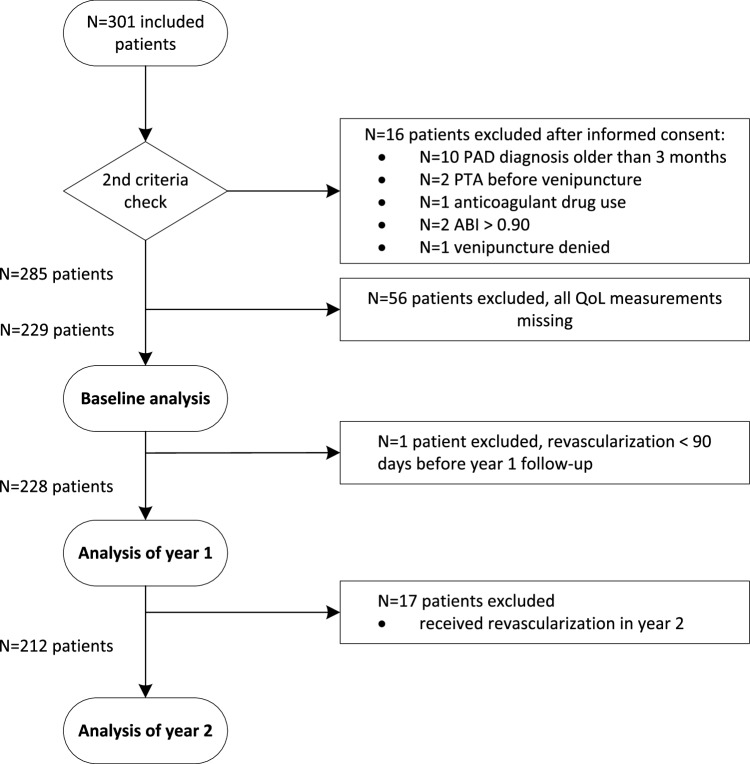
Patient flow

The study population consisted of 285 patients. After exclusion of 56 patients for completely missing QoL measurements, the population analysed consisted of 229 PAD patients (see Fig. 1 for patient flow). Between 16.6 and 42.4% of metrics were missing, the measurement time with the largest proportions of missing values was 1-year follow-up and the metric with the largest proportions of missingness was SF-6D (see Table 5 in Appendix 1). Patients with and without missing QoL scores showed few differences in baseline characteristics (see Table 6 in Appendix 1).

### Population characteristics

**Fig. 2:**
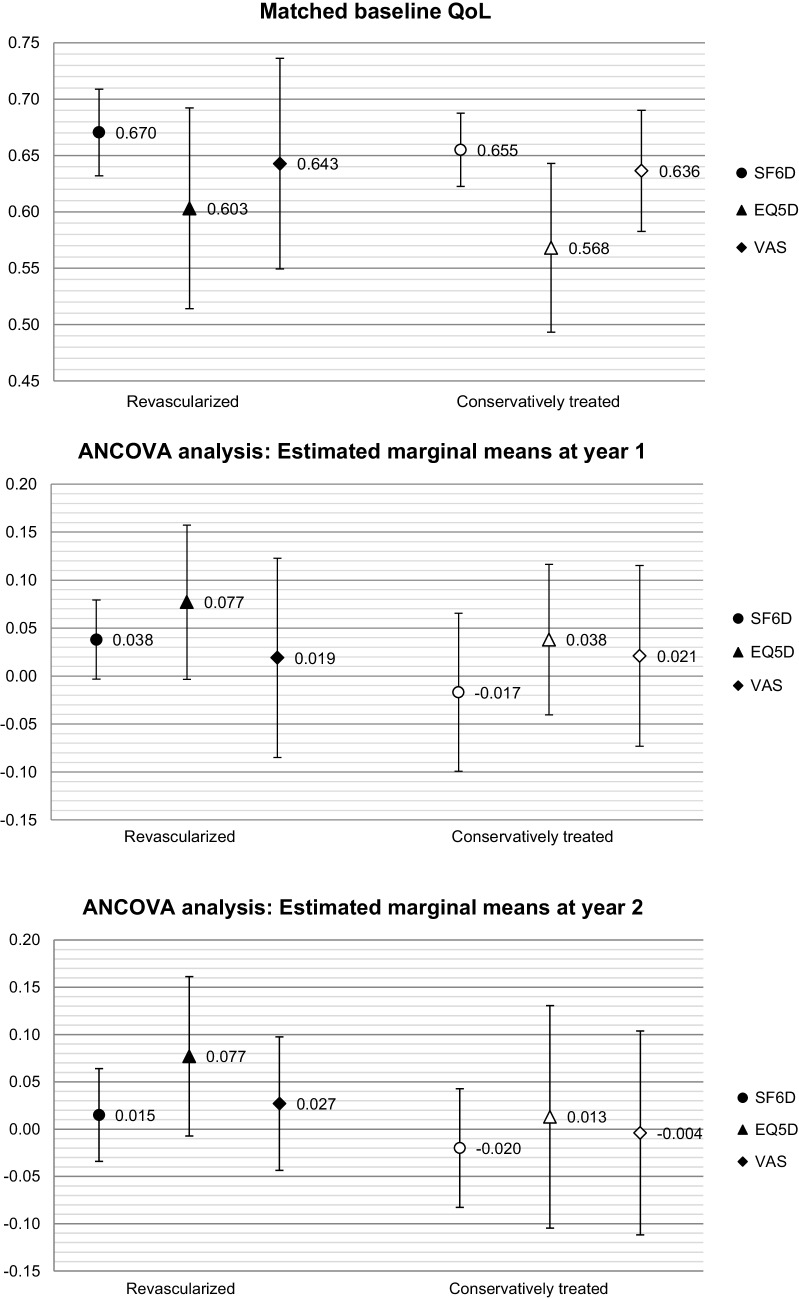
**a–c** Matched baseline scores, EMMs of year 1 and EMMs of year 2

Mean age at baseline was 66 years (SD 8.141), the cohort consisted of 64.6% males and 53.3% current smokers. Mean resting ABI was 0.72 (SD 0.188), the prevalence rates of Fontaine stages IIb, III and IV were 33.6%, 2.2% and 0.9%, respectively (see Table 2 for more baseline patient characteristics). Mean baseline QoL was 0.689 (SE 0.009) measured by the SF-6D, 0.637 (SE 0.019) measured by the EQ-5D and 0.665 (SE 0.015) measured by the VAS. SF-6D and EQ-5D QoL were significantly different from one another, for further details on baseline QoL, see Tables 6 and 7, Figs. 2a and 5 in Appendix 1. At 1-year follow-up, 70 patients (30.6%) had received revascularisation, and no relationship was detected between time since revascularisation and change in QoL at year 1. Eighteen patients (7.9%) experienced a cardiovascular event in the first year and seventeen patients during the second year (7.4%). Seventeen patients were revascularised in the second year (7.4%).

**Table 2:** Baseline characteristics, frequencies and missingness

Characteristic	N^a^ (%)	Missing values (%)
Demographics		
Male gender	148 (64.6)	0 (0)
Currently smoking	122 (53.3)	1 (1)
Age ± SD	65.8 ± 8.1	0 (0)
Body mass index ± SD	26.6 ± 4.1	31 (13)
Disease severity		
Fontaine I	9 (3.9)	1 (1)
Fontaine IIa	136 (59.4)	
Fontaine IIb	77 (33.6)	
Fontaine III	5 (2.2)	
Fontaine IV	2 (0.9)	
Progressive symptoms	116 (50.7)	0 (0)
Rest pain	40 (17.5)	2 (1)
Complaints in daily life	128 (55.9)	5 (2)
Claudication distance < 100 m	61 (26.6)	4 (2)
Ankle-brachial-index ± SD	0.72 ± 0.19	0 (0)
Comorbidities		
Stroke	29 (12.7)	0 (0)
Myocardial infarction	28 (12.2)	0 (0)
No diabetes	190 (83.0)	0 (0)
Untreated diabetes	5 (2.2)	
Diabetes mellitus II	27 (11.8)	
Diabetes mellitus I	7 (3.1)	
Hypertension	121 (54.1)	0 (0)
Cholesterol-lowering drug use	188 (82.1)	0 (0)
Elevated D-Dimer	72 (31.4)	6 (3)
Impaired kidney function	48 (21.0)	30 (13)
Malignancies	21 (9.2)	0 (0)

*SD* standard deviation

^a^Patient characteristics after imputation of missing values

### Revascularisation effect and heterogeneity in response during the first year

**Fig. 3:**
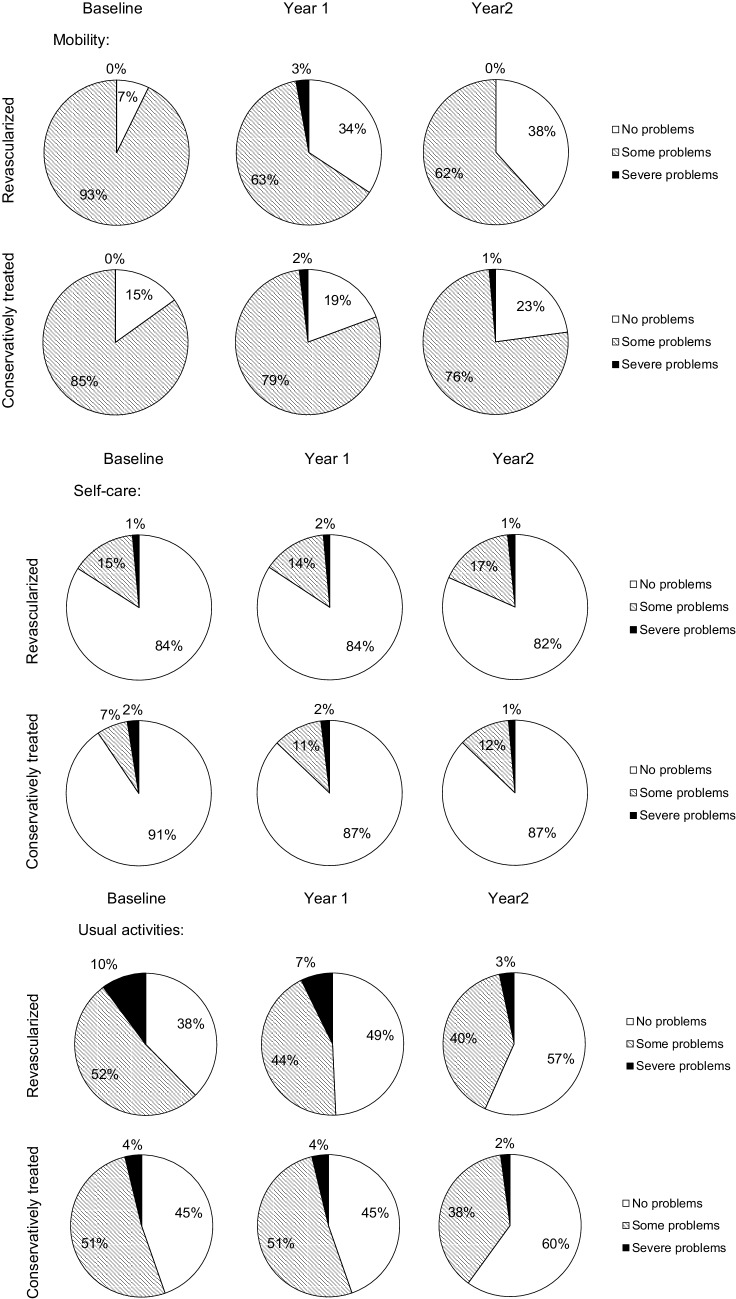

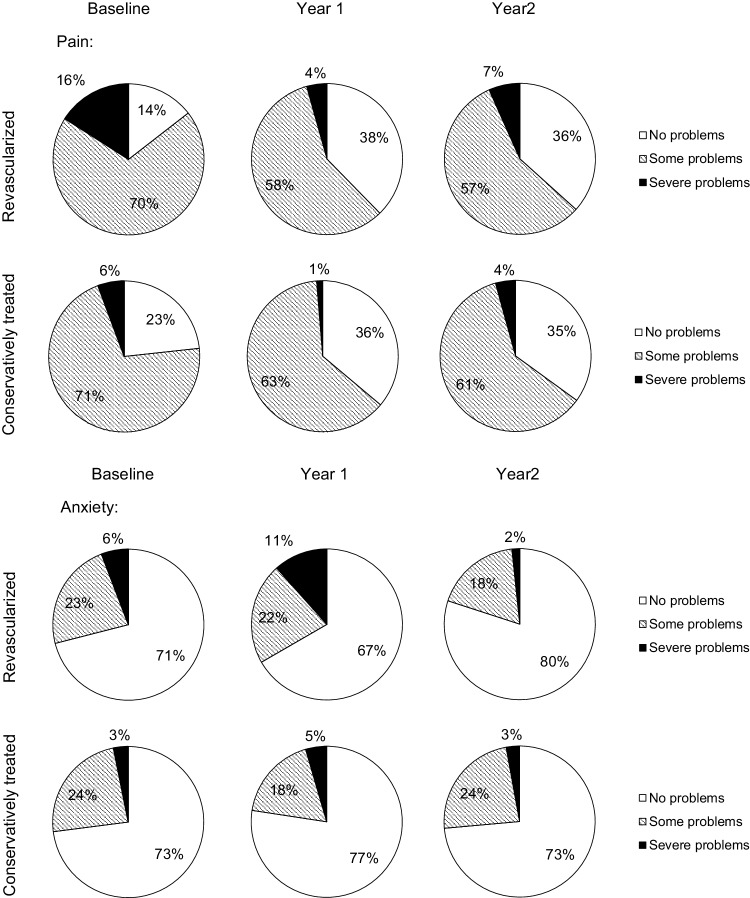
EQ-5D domains over time

**Fig. 4:**
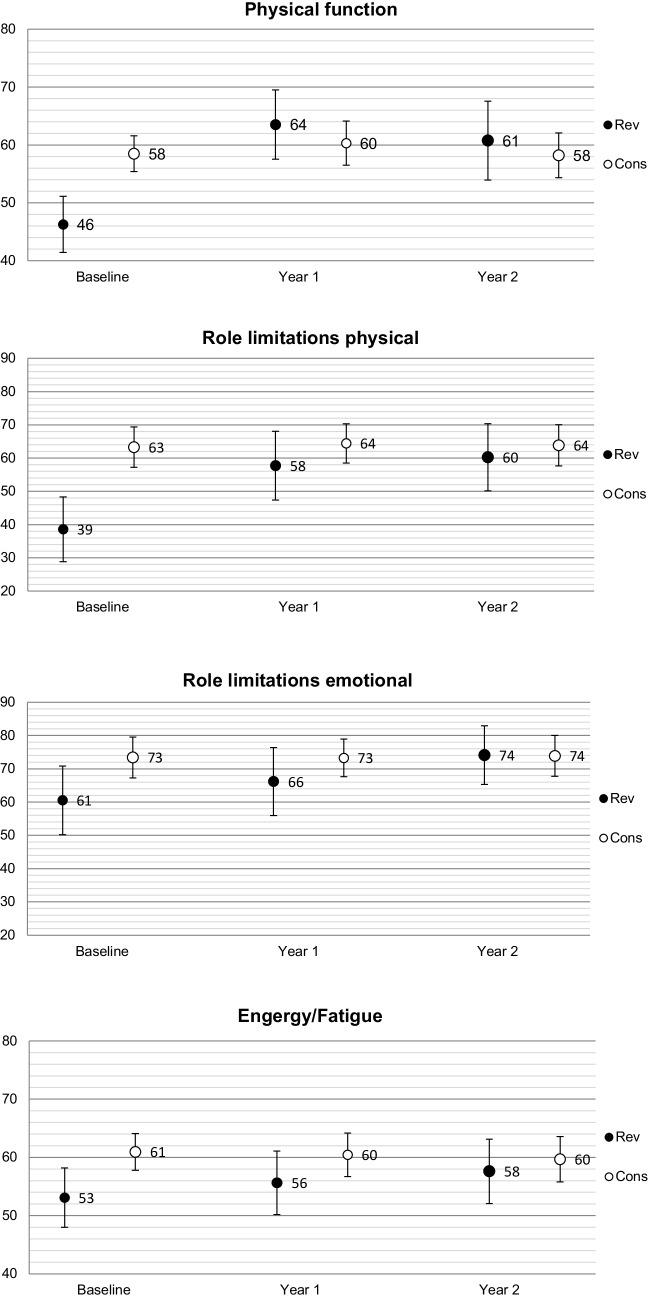

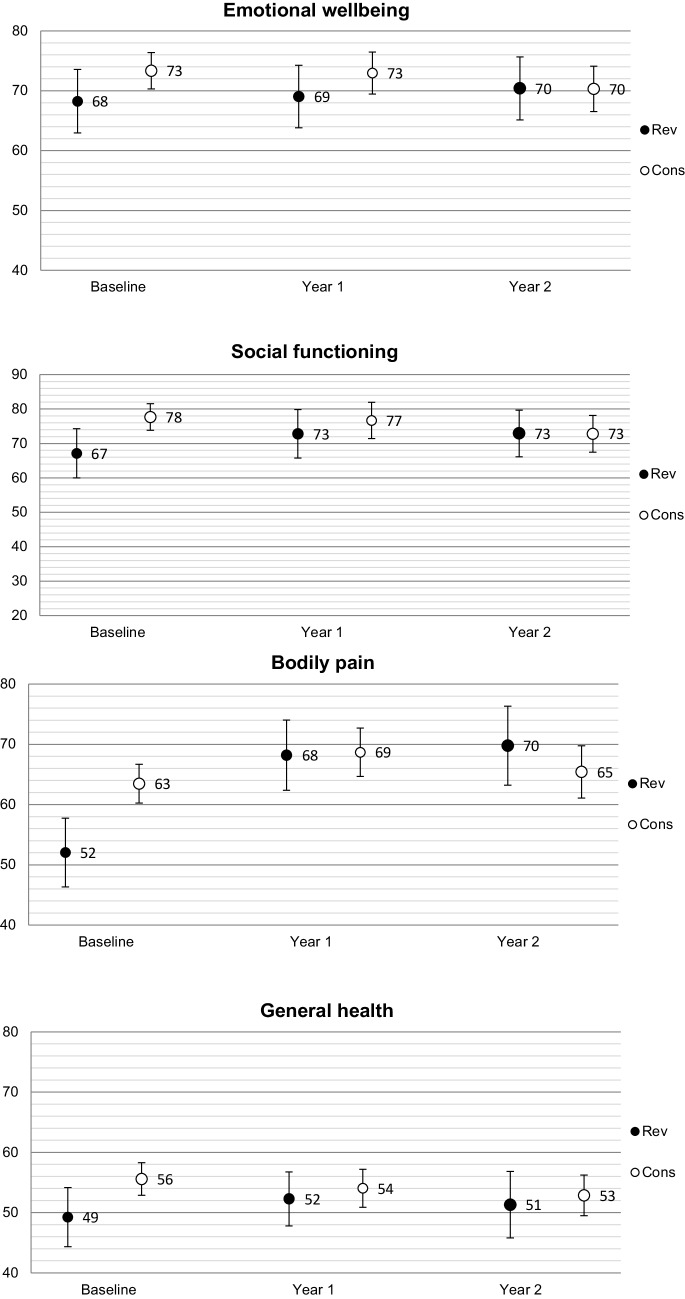
**a–h** SF-36 domain scores over time* *Rev* Revascularized, *Cons* Conservative treatment

The descriptive system of the EQ-5D revealed that QoL gains after revascularisation were driven by increasing proportions of patients reporting ‘no problems’ with pain/discomfort, mobility and usual activities (see Fig. 3 in Appendix 1). All SF-6D domain scores increased, the largest increases were observed in the domains physical functioning, role limitations physical and pain (see Fig. 4 in Appendix 1).

Propensity score matching resulted in improved covariate balance between revascularised and conservatively treated patients. The propensity score and overviews of covariate balance after matching are presented in Appendix 3. Therefore, matched data were used in the ANCOVA analyses. The ANCOVA model (Table 3) showed that baseline QoL is a covariate of QoL change after treatment. All other baseline patient characteristics (see Table 1 for characteristics) and treatment type were not significant covariates. The models indicated QoL gain after treatment was larger in patients with low baseline QoL.

**Table 3:** ANCOVA analysis: coefficients of QoL change baseline – year 1, and baseline-year 2

Model coefficients	SF-6D			EQ-5D			VAS		
	B	SE	Sig.	B	SE	Sig.	B	SE	Sig.
Year 1									
(Intercept)	0.427	0.108	0.001	0.529	0.074	0.000	0.543	0.119	0.000
Conservative treatment	− 0.055	0.042	0.205	− 0.038	0.057	0.506	0.002	0.072	0.978
Baseline SF-6D	− 0.587	0.163	0.001	–	–	–	–	–	–
Baseline EQ-5D	–	–	–	− 0.774	0.119	0.000	–	–	–
Baseline VAS	–	–	–	–	–	–	− 0.817	0.211	0.002
Year 2									
(Intercept)	0.394	0.139	0.008	0.637	0.077	0.000	0.481	0.157	0.007
Conservative treatment	− 0.035	0.038	0.360	− 0.064	0.077	0.416	− 0.031	0.063	0.630
Rest pain	− 0.030	0.047	0.516	− 0.167	0.073	0.026	0.000	0.095	0.996
Baseline SF-6D	− 0.564	0.200	0.008	–	–	–	–	–	–
Baseline EQ-5D	–	–	–	− 0.870	0.117	0.000	–	–	–
Baseline VAS	–	–	–	–	–	–	− 0.713	0.219	0.005

This analysis is based on propensity score-matched data

*B* beta-coefficient, *Sig*. significance, *SE* standard error

Post hoc analyses of the ANCOVA models (Table 4) produced PS-matched EMMs of revascularisation and conservative treatment at year 1. EMMs after revascularisation are consistently positive, while those of conservative treatment are positive and negative (see Fig. 2b in Appendix 1). EMMs of revascularisation and conservative treatment do not differ significantly. Between the metrics, EMMs and mean differences vary in magnitude, EQ-5D EMMs and SF-6D mean differences are largest, VAS EMMs are lowest and the mean difference is negative. Scenario analyses confirm these observations, only the complete case scenario produced scores somewhat different (see Appendix 4).

**Table 4:** ANCOVA post hoc analysis: estimated marginal means of treatment at year 1 and year 2

	Estimated marginal means (SE)			*P* value	*R*^2a^	Adjusted *R*^2a^
	Rev	Cons	Difference			
Year 1						
SF-6D	0.038 (0.021)	− 0.017 (0.042)	0.055 (0.042)	0.205	0.141–0.382	0.128–0.373
EQ-5D	0.077 (0.041)	0.038 (0.040)	0.038 (0.057)	0.506	0.308–0.551	0.398–0.545
VAS	0.019 (0.053)	0.021 (0.048)	− 0.002 (0.072)	0.978	0.227–0.461	0.216–0.453
Year 2						
SF-6D	0.015 (0.025)	− 0.020 (0.032)	0.035 (0.038)	0.360	0.050–0.251	0.026–0.231
EQ-5D	0.077 (0.043)	0.013 (0.060)	0.064 (0.077)	0.416	0.354–0.499	0.338–0.487
VAS	0.027 (0.036)	− 0.004 (0.055)	0.031 (0.063)	0.630	0.059–0.420	0.035–0.405

*Rev* revascularised, *Con* conservative treatment, *SE* standard error

^a^*R*^2^ are presented as ranges due to the presence of multiple imputation datasets

### Sustainability of and heterogeneity in revascularisation effect during the second year

As seen at 1-year follow-up, patients revascularised in year one reported less problems with pain/discomfort, mobility and usual activities in the EQ-5D (see Fig. 3 in Appendix 1). All SF-6D domain scores were increased compared to baseline and year one follow-up except for physical function, which decreased compared to year one follow-up but remained increased compared to baseline (see Fig. 4 in Appendix 1).

Baseline QoL and rest pain are significant covariates of QoL change after treatment, while all other baseline patient characteristics and treatment group were not significant covariates (Table 3). QoL gains after treatment are larger in patients with low baseline QoL, and lower in patients with rest pain.

As at year 1, year 2 EMMs after revascularisation are consistently positive and those of conservative treatment are positive and negative (see Fig. 2c in Appendix 1). Unlike in year 1, all mean differences are positive, yet not statistically significant (Table 4). In comparison to year 1, EMMs of revascularisation were increased, stagnated and decreased measured by SF-6D, EQ-5D and VAS, respectively. Between the metrics, EMMs and mean differences vary in magnitude, the EQ-5D has the largest scores. Scenario analyses also confirm these observations and show similar scores, only the complete case scenario produced scores somewhat different (see Appendix 4).

## Discussion

### Main findings

A year after diagnosis, the effect of revascularisation on QoL is insignificantly positive, and is influenced by baseline QoL. The effect of revascularisation is insignificantly larger than the effect of conservative treatment. Two years after diagnosis, the positive effect of revascularisation on QoL is sustained. Factors influencing the maintained effect of revascularisation on QoL are baseline QoL and rest pain, the latter only on EQ-5D scores. Compared to the first year, a decreased, stable and increased revascularisation effect is depicted by SF-6D, EQ-5D and VAS, respectively. Magnitude of revascularisation effect is generally largest when considering the EQ-5D.

### Interpretation

We found positive effects of revascularisation on QoL at years 1 and 2 measurements. This is in line with literature reporting QoL gains of 0.07 to 0.19 measured with the EQ-5D [10, 37, 38], significant increases in all SF-36 domains [11] and a VAS gain of 0.12 1 year after revascularisation [38]. Moreover, EQ-5D, VAS and SF-36 domain scores 2 years after PAD diagnosis were in line with long-term follow-up scores measured 11 years after revascularisation in van Hattum et al. [39]. Regression analysis had previously shown age, BMI, education, severity of disease and baseline general health to predict SF-36 domain scores 1 year after revascularisation [11, 40]. A different study had found age and diabetes to correlate with SF-36 scores between 1 and 7 years after revascularisation or amputation for PAD; rest pain was tested and found to be insignificant, QoL before the intervention was not tested as a predictor [40]. Differences in patient characteristics, outcome measures and variables in the regression analyses hamper the comparison of these results.

**Fig. 5:**
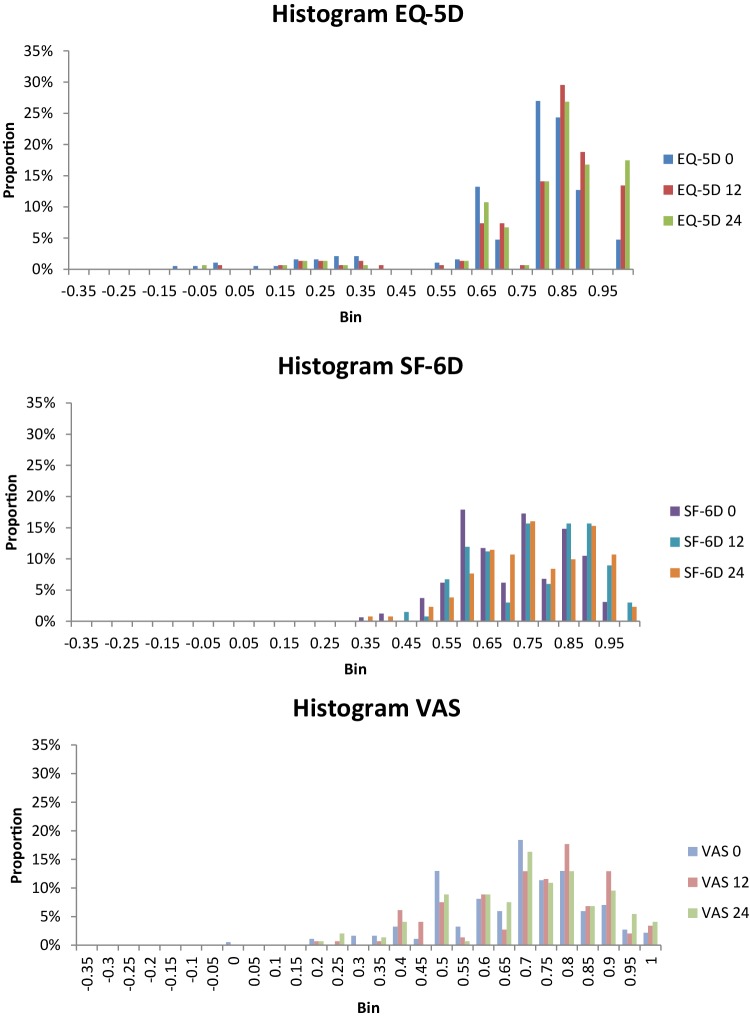
Floor and ceiling effects

As a result of adaptation and coping, patient VAS scores, as estimates of a patient’s own QoL, tend to be higher than EQ-5D scores which reflect the public’s preferences for a patient’s health state description [12, 41, 42]. Our results are in line with these expectations. Furthermore, the mean difference between baseline EQ-5D and SF-6D in our study (EQ-5D 0.052 points larger than SF-6D) was similar to that in other patient populations [43]. The observation that the effect of revascularisation on QoL was larger measured by the EQ-5D might be explained by a floor effect of the SF-6D. The SF-6D, as it was designed to assess QoL in the general population, tends to produce relatively high utility values in patients with a larger disease burden [5, 39]. Figure 5 in Appendix 1 shows that in our sample, values below 0.55 were rare. This floor effect can then cause decreased sensitivity in health states of lower QoL [5, 14, 27, 43–45]. Consistently, it has been hypothesised that QoL valued by the patients themselves have a ceiling effect and reduced discriminative capabilities, which might explain low VAS change scores [12]. Figure 5 in Appendix 1 indicates scores above 0.9 were rare. However, previous studies also identified a potential weakness of the EQ-5D, the overestimation of QoL due to the avoidance of the third and most severe level [29, 43]. In other populations, less than 1% made use of level 3 of the domain ‘mobility’. Avoidance of mobility level 3 can cause an insensitivity of the EQ-5D to improvements in mobility. Figure 3 shows that in our study, only 0–3% of patients responded with level 3 in this domain. Insensitivity to change, however, was not indicated in our results considering mobility was a significant driver of QoL change after treatment. Moreover, a previous literature review concluded the EQ-5D to be more sensitive to change than other generic measures in PAD patients [19], results that we confirmed with the comparatively large estimated marginal means of treatment and the comparatively large difference between treatment groups.

### Strengths and weaknesses

A first strength of this study is the selection of participants; the study population consisting of patients referred to the vascular surgery department for PAD diagnosis reflects the spectrum of PAD patients, including patients with varying medical history and PAD severity. Our outcomes are likely generalisable to PAD patients in secondary care overall. Secondly, by using PS matching, the observational data were resampled to allow for comparisons of revascularised and conservatively treated patients, thereby enabling comparisons of treatment effect. Thirdly, by analysing three widely used QoL metrics, one of them being the current standard in assessing QoL for economic evaluations in, for instance, the Netherlands [28] and the United Kingdom [46], and comparing their scores and performances, this study provides well-needed insight into the strengths and weaknesses as well as the suitability of the metrics for economic evaluations regarding treatment of PAD.

The study also suffered from several limitations. The inclusion time just short of 5 years may have allowed for techniques to evolve over time so that patients might have been exposed to varying treatment methods. Expert opinion indicated these developments were not substantial at the study site. Patients using coagulation-altering medication were excluded. Given these medications will be prescribed for atrial fibrillation, a condition vastly affecting QoL [47–49], the excluded patients might be a subgroup with especially low QoL. As a result, our QoL estimates may be an overestimation of the QoL in the total incident PAD population. Another weakness is that, although this is extremely unlikely given the patients’ long treatment records in the participating hospitals, we cannot rule out that patients could have received revascularisation elsewhere that was not reported. Our research also highlighted several implications for further research. Given the variability of revascularisation effect after accounting for a number of patient characteristics, further research should identify patient characteristics of influence, e.g. socioeconomic determinants such as SES, housing and activity level in daily life, or further PAD-specific determinants such as length and location of the occlusion. The relatively small sample size, especially of revascularised patients, may be a weakness of the study as it may have caused relationships or differences that are present to be statistically insignificant. In this respect, it is important to recall that absence of evidence is not evidence of absence [50]. And lastly, the umbrella term (peripheral) revascularisation summarises a number of interventions aimed at restoring blood flow to the leg. Considering the on-going discussion about patency of endovascular vs. surgical revascularisation [51], further research should compare the sustainability of QoL gains acquired by different revascularisation techniques. Data from randomised controlled trials would furthermore negate the need for propensity score matching as an adjustment for confounding by indication, and would thereby enable stronger conclusions about the comparison of treatments.

## Conclusion

The findings of this study show that conservative and invasive treatment both have a positive effect on QoL, and the effect of invasive treatment is sustained over 2 years. Significance tests show no difference between the treatment options. The results of our analyses confirmed advantages of the EQ-5D in detecting change over time and differences between groups. Our results therefore indicate that EQ-5D utilities may be most suitable for QoL measurement in patients with PAD, and support the preferential application of the EQ-5D in this population. The finding that the magnitude of revascularisation effect is influenced by baseline QoL may be relevant for clinical decision making, as it can give an a priori estimation of the expected QoL gain in individual patients.

## Appendix 1: Additional analyses baseline

See Tables  5, 6 and 7 and Figures 2, 3, 4 and 5.

**Table 5:** Available and missing data, scores at floor and ceiling

	Baseline	1-Year follow-up	2-Year follow-up
N	229	225	218
At least one QoL score available	91.3%	66.8%	69.4%
SF-6D missing	28.4%	41.5%	42.4%
EQ-5D missing	16.6%	35.4%	33.6%
VAS missing	17.5%	35.8%	34.1%

**Table 6:** Characteristics of patients with and without missing QoL measurements

Characteristic	Baseline					Year 1		Year 2	
	Complete cohort (229)	Rev. (70)	Cons. (159)	All instruments completed (142)	One or more missing (87)	All instruments completed (127)	One or more missing (98)	All instruments completed (127)	One or more missing (91)
SF-6D (mean (SE))	0.689 (0.009)	0.651 (0.015)	0.706 (0.010)	0.710 (0.011)	0.655 (0.015)	**0.748** (0.011)	**0.654** (0.013)	**0.746** (0.012)	**0.657** (0.013)
EQ-5D (mean (SE))	0.637 (0.019)	0.571 (0.036)	0.666 (0.020)	0.664 (0.020)	0.594 (0.037)	**0.729** (0.018)	**0.623** (0.028)	0.738 (0.019)	0.667 (0.028)
VAS (mean (SE))	0.665 (0.015)	0.629 (0.029)	0.681 (0.018)	0.684 (0.013)	0.633 (0.032)	0.711 (0.016)	0.654 (0.040)	0.712 (0.016)	0.675 (0.039)
Age (mean)	65.8	64.0	66.5	65.3	66.5	66.0	65.7	64.9	66.8
Men (%)	64.6	67.1	63.5	68.3	58.6	69.3	57.1	72.4	54.9
ABI (mean)	0.72	0.70	0.73	0.71	0.73	0.71	0.73	0.69	0.76
Current smoker (%)	53.3	58.6	50.9	55.6	49.4	54.3	53.1	58.3	47.1
Severe Fontaine stage (%)	36.7	51.4	30.2	32.4	43.7	31.5	43.9	29.9	45.1
Progressive symptoms (%)	50.7	74.3	40.3	51.4	49.4	48.8	52.0	48.8	52.9
Hypertension (%)	54.1	51.4	53.5	53.5	51.7	52.0	57.1	50.4	55.9
Hypercholesterolemia (%)	49.8	45.7	51.6	46.5	55.2	52.0	48.0	52.0	47.1
Diabetes (%)	17.0	15.7	17.6	16.9	17.2	16.5	15.3	15.7	18.6
Myocardial infarction (%)	12.2	7.1	14.5	11.3	13.8	8.6	16.3	10.2	14.7
Stroke (%)	12.7	17.1	10.7	12.7	12.6	12.6	11.2	12.7	11.8

Significantly different utilities marked bold

*Rev* revascularisation procedure, including endovascular interventions, e.g. angioplasty with and without stent placement, and open surgery, e.g. atherectomy and endarterectomy, and bypass surgery, *Cons* conservative treatment, *SE* standard error

**Table 7:** Baseline heterogeneity in quality of life

Characteristic	SF-6D			EQ-5D			VAS		
	Mean	(SE)	Median^a^	Mean	(SE)	Median^a^	Mean	(SE)	Median^a^
Demographics									
Male gender	0.692	0.011	0.696	0.632	0.022	0.691	0.663	0.022	0.700
Female gender	0.684	0.014	0.673	0.647	0.030	0.691	0.668	0.024	0.700
Currently smoking	0.671	0.012	0.666	0.619	0.024	0.691	0.650	0.022	0.700
Currently not smoking	0.710	0.013	0.704	0.658	0.027	0.726	0.682	0.026	0.700
Age > 75	0.672	0.024	0.683	0.633	0.053	0.691	0.651	0.048	0.700
Age < 75	0.692	0.010	0.696	0.638	0.020	0.691	0.667	0.019	0.700
Body mass index > 30	0.657	0.018	0.642	0.596	0.047	0.691	0.626	0.037	0.640
Body mass index < 30	0.696	0.010	0.696	0.647	0.019	0.691	0.674	0.016	0.700
Disease severity									
Fontaine mild	0.704	0.011	0.700	0.670	0.020	0.691	0.679	0.019	0.700
Fontaine severe	0.664	0.015	0.645	0.581	0.033	0.691	0.641	0.027	0.700
Progressive symptoms	0.662	0.013	0.649	0.587	0.026	0.691	0.639	0.020	0.700
Non-progressive symptoms	0.717	0.012	0.728	0.689	0.023	0.691	0.692	0.021	0.700
Rest pain	0.626	0.017	0.614	0.558	0.039	0.620	0.587	0.035	0.600
No Rest pain	0.703	0.010	0.699	0.654	0.022	0.691	0.681	0.016	0.700
Complaints in daily life	0.646	0.011	0.630	0.578	0.025	0.691	0.626	0.020	0.650
No complaints in daily life	0.744	0.012	0.753	0.713	0.022	0.727	0.715	0.024	0.750
Claudication < 100 m walking	0.664	0.018	0.646	0.548	0.037	0.691	0.608	0.031	0.640
Claudication > 100 m walking	0.698	0.010	0.696	0.670	0.021	0.691	0.686	0.018	0.700
Ankle-brachial-index < 0.5	0.703	0.028	0.753	0.622	0.056	0.691	0.688	0.049	0.700
Ankle-brachial-index > 0.9	0.688	0.010	0.675	0.639	0.019	0.691	0.662	0.016	0.700
Comorbidities									
Stroke	0.661	0.024	0.648	0.557	0.053	0.691	0.622	0.046	0.600
No stroke	0.693	0.010	0.677	0.649	0.020	0.691	0.671	0.015	0.700
Myocardial infarction	0.701	0.023	0.698	0.661	0.049	0.691	0.677	0.061	0.700
No myocardial infarction	0.688	0.010	0.675	0.634	0.020	0.691	0.663	0.015	0.700
Diabetes	0.674	0.020	0.669	0.631	0.044	0.691	0.628	0.037	0.675
No diabetes	0.692	0.010	0.677	0.639	0.021	0.691	0.673	0.016	0.700
Hypertension	0.688	0.012	0.640	0.640	0.022	0.691	0.656	0.021	0.700
No hypertension	0.691	0.013	0.635	0.635	0.029	0.691	0.674	0.022	0.700
Cholesterol-lowering drug use	0.693	0.010	0.691	0.635	0.022	0.691	0.664	0.017	0.700
No Cholesterol-lowering drug use	0.671	0.020	0.637	0.649	0.032	0.691	0.669	0.034	0.700
Elevated D-Dimer^b^	0.670	0.015	0.671	0.615	0.030	0.691	0.649	0.028	0.700
Normal D-Dimer	0.698	0.011	0.694	0.648	0.023	0.691	0.672	0.018	0.700
Impaired kidney function^c^	0.705	0.017	0.698	0.699	0.030	0.727	0.679	0.039	0.720
Normal kidney function	0.685	0.010	0.673	0.621	0.022	0.691	0.661	0.017	0.700
Malignancies	0.730	0.025	0.753	0.699	0.052	0.727	0.665	0.068	0.700
No malignancies	0.685	0.009	0.675	0.631	0.019	0.691	0.665	0.016	0.700

*SE* standard error

^a^Median of all 10 imputed datasets combined

^b^Elevated D-Dimer is defined as D-Dimer > 500 when age < 50, as D-Dimer > age * 10 when age > 50

^c^Impaired kidney function is defined as MDRD eGFR below 60

## Appendix 2: Additional information

List of exclusion criteria:

- PAD diagnosis more than 3 months prior to study inclusion,
- cardiovascular or arterial interventions within the past 6 months,
- (unstable) angina pectoris, myocardial infarction, stroke or heart failure within the past 3 months,
- known coagulation disorders,
- anticoagulant medication use (e.g. Vitamin K antagonists, direct factor Xa-inhibitors and factor II-inhibitors, heparin),
- chronic inflammatory diseases,
- active malignancies,
- repeatedly failed venipunctures,
- being underage,
- not meeting the inclusion criteria.

List of events summarised in the term cardiovascular events:

- transient ischemic attack,
- stroke,
- other cerebral events,
- angina pectoris,
- myocardial infarction,
- other ischemic events,
- coronary revascularisation,
- abdominal aortic aneurysm,
- other artery diseases.

## Appendix 3: Propensity score matching

See Table 8.

List of parameters included in the propensity score:

- Progressive symptoms;
- Complaints in daily life;
- Claudication distance;
- Baseline SF-6D;
- Domains of the SF-36:
  - Physical Function;
  - Limitations Physical;
  - Bodily Pain;
- Age;
- Myocardial infarction;
- Currently smoking.

**Table 8:** Group characteristics after propensity score matching for year 1

Characteristic	Pre-matching pooled		Post-matching pooled	
	Rev	Cons	Rev	Cons
Quality of life				
SF-6D baseline	0.65	0.71	0.66	0.67
EQ-5D baseline	0.58	0.67	0.57	0.60
VAS baseline	0.63	0.69	0.64	0.64
Demographics				
Male gender (%)	67.14	63.52	65.48	67.33
Currently smoking (%)	58.57	50.82	58.24	57.95
Age (± SD)	63.97	66.53	64.76	64.44
Body mass index (± SD)	26.11	26.76	25.88	27.24
Disease severity				
Fontaine I (%)	1.43	5.03	1.42	4.40
Fontaine IIa (%)	47.14	64.84	49.72	55.40
Fontaine IIb (%)	45.71	28.49	43.04	38.21
Fontaine III (%)	2.86	1.51	2.84	1.99
Fontaine IV (%)	2.86	0.13	2.98	0.00
Progressive symptoms (%)	74.29	40.25	71.45	75.00
Rest pain (%)	31.71	11.07	29.40	17.19
Complaints in daily life (%)	75.00	47.36	74.29	73.15
Claudication distance < 100 m (%)	45.29	18.36	42.61	44.89
Ankle brachial index (± SD)	0.70	0.73	0.70	0.71
Comorbidities				
Stroke (%)	17.14	10.69	17.61	16.48
Myocardial infarction (%)	7.14	14.47	7.95	9.23
No diabetes (%)	84.29	82.39	85.37	77.70
Untreated diabetes (%)	1.43	2.52	1.42	8.66
Diabetes mellitus II (%)	14.29	10.69	13.21	9.52
Diabetes mellitus I (%)	0.00	4.40	0.00	4.12
Hypertension (%)	52.86	54.72	50.85	59.80
Cholesterol-lowering drug use (%)	84.29	81.13	84.23	79.83
Elevated D-Dimer (%)	32.86	30.82	32.53	30.82
Impaired kidney function (%)	12.86	24.53	13.78	14.06
Malignancies (%)	7.14	10.06	7.39	6.39

*Rev* revascularised, *Cons* conservatively treated, *SD* standard deviation

## Appendix 4: Scenario analyses

See Tables 9, 10, 11, 12, 13, 14, 15, 16, 17 and 18.

### Year 1

**Table 9:** Crude scores with propensity score matching, QoL change baseline to year 1

	Rev crude score (SE)	Cons crude score (SE)	Mean difference (SE)	*P* value
SF-6D	0.042 (0.022)	− 0.021 (0.043)	0.063 (0.043)	0.158
EQ-5D	0.089 (0.047)	0.026 (0.050)	0.064 (0.071)	0.377
VAS	0.023 (0.054)	0.018 (0.059)	0.005 (0.082)	0.951

*Rev* revascularized, *Cons* conservative treatment

#### Scenario 1: Unmatched sample

**Table 10:** EMMs without propensity score matching, QoL change baseline to year 1

	Rev EMM (SE)	Cons EMM (SE)	Mean difference (SE)	*P* value	*R*^2^	Adjusted *R*^2^
SF-6D	0.027 (0.020)	− 0.004 (0.012)	0.031 (0.022)	0.155	0.176–0.228	0.168–0.222
EQ5D	0.038 (0.035)	0.030 (0.025)	0.008 (0.041)	0.842	0.259–0.438	0.252–0.433
VAS	0.003 (0.045)	0.013 (0.020)	− 0.010 (0.051)	0.851	0.264–0.410	0.257–0.405

	Rev crude score (SE)	Cons crude score (SE)	Mean difference (SE)	*P* value
SF-6D	0.049 (0.020)	− 0.013 (0.014)	0.062 (0.023)	0.008
EQ-5D	0.090 (0.046)	0.008 (0.026)	0.083 (0.047)	0.084
VAS	0.034 (0.048)	− 0.001 (0.025)	0.035 (0.055)	0.527

*R*^2^ are presented as ranges due to the presence of multiple imputation datasets

*Rev* revascularised, *Cons* conservative treatment, *EMM* estimated marginal mean

#### Scenario 2: Unmatched sample, exclusively using patients without cardiovascular events during follow-up

**Table 11:** EMMs without propensity score matching, QoL change baseline to year 1

	Rev EMM (SE)	Cons EMM (SE)	Mean difference (SE)	*P* value	*R*^2^	Adjusted *R*^2^
SF-6D	0.033 (0.019)	0.006 (0.012)	0.027 (0.021)	0.199	0.215–0.252	0.207–0.244
EQ-5D	0.031 (0.035)	0.031 (0.026)	0.000 (0.042)	0.992	0.254–0.447	0.247–0.442
VAS	0.006 (0.046)	0.027 (0.020)	− 0.020 (0.053)	0.703	0.310–0.459	0.304–0.454

	Rev crude score (SE)	Cons crude score (SE)	Mean difference (SE)	*P* value
SF-6D	0.057 (0.021)	− 0.004 (0.013)	0.060 (0.023)	0.009
EQ-5D	0.087 (0.048)	0.008 (0.027)	0.079 (0.052)	0.132
VAS	0.049 (0.050)	0.009 (0.025)	0.041 (0.056)	0.477

*R*^2^ are presented as ranges due to the presence of multiple imputation datasets

*Rev* revascularised, *Cons* conservative treatment, *EMM* estimated marginal mean

#### Scenario 3: Unmatched sample, exclusively using complete cases

**Table 12:** EMMs without propensity score matching, QoL change baseline to year 1

	Rev EMM (SE)	Cons EMM (SE)	Mean difference (SE)	*P* value	*R*^2^	Adjusted *R*^2^
SF-6D	0.052 (0.023)	0.021 (0.016)	0.031 (0.029)	0.274	0.102	0.084
EQ-5D	0.045 (0.029)	0.030 (0.020)	0.015 (0.036)	0.671	0.333	0.322
VAS	− 0.010 (0.027)	0.003 (0.019)	− 0.014 (0.033)	0.686	0.126	0.113

	Rev crude score (SE)	Cons crude score (SE)	Mean difference (SE)	*P* value
SF-6D	0.066 (0.026)	0.015 (0.015)	0.042 (0.025)	0.073
EQ-5D	0.085 (0.040)	0.012 (0.022)	0.053 (0.043)	0.111
VAS	0.007 (0.035)	− 0.005 (0.018)	0.009 (0.031)	0.726

*R*^2^ are presented as ranges due to the presence of multiple imputation datasets

*Rev* revascularised, *Cons* conservative treatment, *EMM* estimated marginal mean

#### Scenario 4: Matched sample, excluding patients revascularised in the second half of the first follow-up year

**Table 13:** EMMs with propensity score matching, QoL change baseline to year 1

	Rev EMM (SE)	Cons EMM (SE)	Mean difference (SE)	*P* value	*R*^2^	Adjusted *R*^2^
SF-6D	0.015 (0.025)	− 0.020 (0.032)	0.035 (0.038)	0.360	0.050–0.251	0.026–0.231
EQ-5D	0.077 (0.043)	0.013 (0.060)	0.064 (0.077)	0.416	0.354–0.499	0.338–0.487
VAS	0.027 (0.036)	− 0.004 (0.055)	0.031 (0.063)	0.630	0.059–0.420	0.035–0.405

	Rev crude score (SE)	Cons crude score (SE)	Mean difference (SE)	*P* value
SF-6D	0.018 (0.026)	− 0.023 (0.032)	0.040 (0.038)	0.292
EQ-5D	0.083 (0.054)	0.007 (0.068)	0.076 (0.092)	0.417
VAS	0.037 (0.054)	− 0.014 (0.064)	0.050 (0.077)	0.519

*R*^2^ are presented as ranges due to the presence of multiple imputation datasets

*Rev* revascularised, *Cons* conservative treatment, *EMM* estimated marginal mean

### Year 2

**Table 14:** EMMs with propensity score matching, QoL change baseline to year 2

	Rev crude score (SE)	Cons crude score (SE)	Mean difference (SE)	*P* value
SF-6D	0.018 (0.026)	− 0.023 (0.032)	0.040 (0.038)	0.292
EQ-5D	0.083 (0.054)	0.007 (0.068)	0.076 (0.092)	0.417
VAS	0.037 (0.054)	− 0.014 (0.064)	0.050 (0.077)	0.519

*Rev* revascularised, *Cons* conservative treatment, *EMM* estimated marginal mean

#### Scenario 1: Unmatched sample

**Table 15:** EMMs without propensity score matching, QoL change baseline to year 2

	Rev EMM (SE)	Cons EMM (SE)	Mean difference (SE)	*P* value	*R*^2^	Adjusted *R*^2^
SF-6D	0.016 (0.025)	− 0.035 (0.016)	0.050 (0.030)	0.093	0.078–0.108	0.065–0.095
EQ-5D	0.068 (0.038)	0.011 (0.023)	0.057 (0.044)	0.198	0.301–0.435	0.291–0.427
VAS	0.019 (0.038)	− 0.015 (0.026)	0.034 (0.045)	0.448	0.164–0.307	0.152–0.297

	Rev rude score (SE)	Cons crude score (SE)	Mean difference (SE)	*P* value
SF-6D	0.051 (0.020)	− 0.015 (0.014)	0.067 (0.025)	0.007
EQ-5D	0.084 (0.047)	0.010 (0.028)	0.074 (0.049)	0.136
VAS	0.040 (0.046)	− 0.002 (0.026)	0.042 (0.053)	0.433

*R*^2^ are presented as ranges due to the presence of multiple imputation datasets

*Rev* revascularised, *Cons* conservative treatment, *EMM* estimated marginal mean

#### Scenario 2: Unmatched sample, exclusively using patients without cardiovascular events during follow-up

**Table 16:** EMMs without propensity score matching, QoL change baseline to year 2

	Rev EMM (SE)	Cons EMM (SE)	Mean difference (SE)	*P* value	*R*^2^	Adjusted *R*^2^
SF-6D	0.021 (0.025)	− 0.021 (0.017)	0.041 (0.030)	0.167	0.074–0.102	0.058–0.087
EQ-5D	0.062 (0.043)	0.015 (0.024)	0.048 (0.049)	0.327	0.304–0.452	0.292–0.443
VAS	0.021 (0.041)	− 0.012 (0.028)	0.033 (0.047)	0.476	0.156–0.341	0.141–0.330

	Rev crude score (SE)	Cons crude score (SE)	Mean difference (SE)	*P* value
SF-6D	0.055 (0.022)	0.000 (0.014)	0.055 (0.025)	0.031
EQ-5D	0.072 (0.053)	0.012 (0.030)	0.060 (0.054)	0.270
VAS	0.043 (0.051)	0.005 (0.026)	0.037 (0.058)	0.523

*R*^2^ are presented as ranges due to the presence of multiple imputation datasets

*Rev* revascularised, *Cons* conservative treatment, *EMM* estimated marginal mean

#### Scenario 3: Unmatched sample, exclusively using complete cases

**Table 17:** EMMs without propensity score matching, QoL change baseline to year 2

	Rev EMM (SE)	Cons EMM (SE)	Mean difference (SE)	*P* value	*R*^2^	Adjusted *R*^2^
SF-6D	0.001 (0.033)	0.011 (0.021)	− 0.010 (0.040)	0.800	0.075	0.045
EQ-5D	0.031 (0.033)	0.038 (0.023)	− 0.007 (0.040)	0.863	0.391	0.376
VAS	0.005 (0.027)	− 0.011 (0.021)	0.016 (0.035)	0.643	0.060	0.036

	Rev crude score (SE)	Cons crude score (SE)	Mean difference (SE)	*P* value
SF-6D	0.012 (0.046)	0.009 (0.016)	0.003 (0.048)	0.951
EQ-5D	0.059 (0.048)	0.028 (0.025)	0.031 (0.054)	0.563
VAS	0.010 (0.032)	− 0.015 (0.019)	0.024 (0.035)	0.482

*R*^2^ are presented as ranges due to the presence of multiple imputation datasets

*Rev* revascularised, *Cons* conservative treatment, *EMM* estimated marginal mean

#### Scenario 4: Matched sample, excluding patients revascularised in the second half of the first follow-up year

**Table 18:** EMMs with propensity score matching, QoL change baseline to year 2

	Rev EMM (SE)	Cons EMM (SE)	Mean difference (SE)	*P* value	*R*^2^	Adjusted *R*^2^
SF-6D	0.015 (0.025)	− 0.020 (0.032)	0.035 (0.038)	0.360	0.050–0.251	0.026–0.231
EQ-5D	0.077 (0.043)	0.013 (0.060)	0.064 (0.077)	0.416	0.354–0.499	0.338–0.487
VAS	0.027 (0.036)	− 0.004 (0.055)	0.031 (0.063)	0.630	0.059–0.420	0.035–0.405

	Rev crude score (SE)	Cons crude score (SE)	Mean difference (SE)	*P* value
SF-6D	0.018 (0.026)	− 0.023 (0.032)	0.040 (0.038)	0.292
EQ-5D	0.083 (0.054)	0.007 (0.068)	0.076 (0.092)	0.417
VAS	0.037 (0.054)	− 0.014 (0.064)	0.050 (0.077)	0.519

*R*^2^ are presented as ranges due to the presence of multiple imputation datasets

*Rev* revascularised, *Cons* conservative treatment, *EMM* estimated marginal mean
